# High-quality genome assembly and linkage map for a rapidly evolving plant species: *Silene uniflora*

**DOI:** 10.1093/g3journal/jkag002

**Published:** 2026-01-13

**Authors:** Owen G Osborne, Daniel P Wood, Mariya P Dobreva, Luke T Dunning, Rachel Tucker, Sarah E R Coates, Jaume Pellicer, Jon Holmberg, Adam C Algar, Greta Bocedi, Cecile Gubry-Rangin, Leonel Herrera-Alsina, Berry Juliandi, Lesley T Lancaster, Pascal Touzet, Justin M J Travis, Alexander S T Papadopulos

**Affiliations:** Molecular Ecology and Evolution Group, School of Environmental and Natural Sciences, Bangor University, Bangor LL57 2DG, United Kingdom; Molecular Ecology and Evolution Group, School of Environmental and Natural Sciences, Bangor University, Bangor LL57 2DG, United Kingdom; Royal Botanic Gardens, Kew, Richmond TW9 3AB, United Kingdom; Molecular Ecology and Evolution Group, School of Environmental and Natural Sciences, Bangor University, Bangor LL57 2DG, United Kingdom; Ecology and Evolutionary Biology, School of Biosciences, Sheffield S10 2TN, United Kingdom; Ecology and Evolutionary Biology, School of Biosciences, Sheffield S10 2TN, United Kingdom; Molecular Ecology and Evolution Group, School of Environmental and Natural Sciences, Bangor University, Bangor LL57 2DG, United Kingdom; Royal Botanic Gardens, Kew, Richmond TW9 3AB, United Kingdom; Royal Botanic Gardens, Kew, Richmond TW9 3AB, United Kingdom; Molecular Ecology and Evolution Group, School of Environmental and Natural Sciences, Bangor University, Bangor LL57 2DG, United Kingdom; Department of Biology, Lakehead University, Thunder Bay, Ontario P7B 5E1, Canada; School of Biological Sciences, University of Aberdeen, Aberdeen AB24 2TZ, United Kingdom; School of Biological Sciences, University of Aberdeen, Aberdeen AB24 2TZ, United Kingdom; School of Biological Sciences, University of Aberdeen, Aberdeen AB24 2TZ, United Kingdom; School of Mathematical & Statistical Sciences, University of Galway, Galway H91 TK33, Ireland; Department of Biology, Faculty of Mathematics and Natural Sciences, IPB University, Bogor 16680, West Java, Indonesia; School of Biological Sciences, University of Aberdeen, Aberdeen AB24 2TZ, United Kingdom; Univ Lille, CNRS, UMR 8198—Evo-Eco-Paleo, Lille F-59000, France; School of Biological Sciences, University of Aberdeen, Aberdeen AB24 2TZ, United Kingdom; Molecular Ecology and Evolution Group, School of Environmental and Natural Sciences, Bangor University, Bangor LL57 2DG, United Kingdom

**Keywords:** *Silene*, Caryophyllaceae, campion, sex chromosomes, genomic rearrangements, genome assembly

## Abstract

The genus *Silene* is an important model system for fields as diverse as sex chromosome evolution, speciation, and disease ecology. However, genomic resources remain scarce in the genus. Here, we present a near chromosome-scale genome assembly and high-density linkage map for *S. uniflora*, a hermaphroditic/gynodioecious species which is an important model for rapid adaptation to anthropogenic disturbance and the role of phenotypic plasticity in adaptive evolution. Using a combination of long-read and Hi-C sequencing technologies, we generated a 1,268 Mb genome assembly with a scaffold N50 of 40.72 Mb and 682 Mb assembled into 12 chromosomes. We annotated the genome using evidence from transcriptome and protein mapping in combination with *ab initio* gene prediction, resulting in 41,603 protein-coding genes and a BUSCO completeness score of 91%. We also present a linkage map which we used to validate the genome assembly and estimate local recombination rate across the genome. Comparison to the only 2 other *Silene* species with chromosome-scale genome assemblies reveals widespread genome rearrangements in the genus, suggesting *Silene* may be a promising study system for the role of genome rearrangement in evolution, particularly in the evolution of sex chromosomes and adaptation.

## Introduction

Sea campion, *Silene uniflora*, is a perennial herb native to coastal Northern and Western Europe (Davy et al. 2024). Morphological and genetic studies of *S. uniflora* and its close relative *S. vulgaris* in the mid-20th century were fundamental to the development of the field of experimental taxonomy (Marsden-Jones and Turrill 1957; Davy et al. 2024). Subsequently, it became a model species for the study of castrating sexually transmitted fungal diseases (Chung et al. 2012; Abbate et al. 2018). It has also attracted attention for its rapid adaptation to the extreme environments resulting from non-ferrous metal mining (Baker 1974, 1978; Baker and Dalby 1980). More recently, the species has become an emerging model system for research into the genetic basis of parallel evolution (Papadopulos et al. 2021) and the role of plasticity and gene expression during rapid adaptation (Wood et al. 2023; Coates et al. 2025). More broadly, *Silene* has been the focus of intense research to understand sex chromosome evolution (Papadopulos et al. 2015; Yue et al. 2023), cytonuclear incompatibilities (Garraud et al. 2011; Postel et al. 2022), the evolution of mitochondrial genomes (Sloan et al. 2012; Wu et al. 2015), biological invasions (Keller and Taylor 2010; Jenkins and Keller 2011; Castillo et al. 2014), adaptation, and speciation (Bratteler et al. 2006; Muir et al. 2012; Favre et al. 2017; Zemp et al. 2018). Nevertheless, the first chromosome-scale assembly was published only recently (Fields et al. 2023).

There are currently only high-quality reference genomes available for 2 *Silene* species—*S. latifolia* and *S. conica* (Fields et al. 2023; Yue et al. 2023). *S. latifolia* is dioecious and has been the focus of intense research into the evolution of young, homomorphic sex chromosomes. The majority of *Silene* species are diploid with 12 pairs of chromosomes (2*n* = 24, including *S. latifolia* and *S. uniflora*; (Bari 1973). *S. conica* is purely hermaphroditic and is unusual as it only possesses ten chromosome pairs (2*n* = 20; (Fields et al. 2023). *S. latifolia* and *S. conica* also sit at the extreme ends of genome size for *Silene* species—*S. latifolia* has one of the largest diploid genome sizes (2.8 Gb; (Pellicer and Leitch 2020), whereas *S. conica* is one of the smallest (0.9 Gb; (Williams et al. 2021). Populations of *S. uniflora* can exclusively contain hermaphrodites or be gynodioecious (Davy et al. 2024). Its genome size is intermediate for the genus (1.2 Gb), it has 12 pairs of chromosomes (Williams et al. 2021) and B chromosomes have also been observed in karyotyping studies (Cobon and Murray 1983). High-quality genomic resources for the species have the potential to accelerate research into the genomic and epigenetic mechanisms that underlie rapid adaptation but also contribute to wider research in the genus by adding additional resources for studies of sex chromosomes and mating systems. Here, we report a near chromosome-level genome assembly and a detailed genetic map for *S. uniflora*, providing a comprehensive resource for future eco-evolutionary research.

## Materials and methods

### Plant material and sequence data generation

Cuttings from a single coastal individual were collected in Tresaith (West Wales, UK) and propagated and self-fertilized as part of a previous study (Papadopulos et al. 2021). A single individual inbred F1 (SUTF1P; draft genome ASM1898310v1) was selfed and the F2 individual sequenced here was grown under controlled conditions at the Henfaes Research Centre, Bangor University, UK. High molecular weight DNA was extracted for Pacific Biosciences (PacBio) and Oxford Nanopore (ONT) sequencing using a Qiagen DNAeasy Maxi plant kit. PacBio library preparation and sequencing was completed at the Genomics Laboratory, University of Sheffield, UK (Supplementary note 1). Liquid nitrogen frozen leaf tissue was sent to Dovetail Genomics, LLC for Hi-C, Chicago and Illumina Truseq library preparation and sequencing on a HiSeqX sequencer (Illumina).

RNA extraction was conducted on root, flower, and leaf tissue for the genome plant using the RNeasy Plant Mini Kit (Qiagen), including a DNase digestion step, and RNA extracts were sent to the Beijing Genomics Institute (Hong Kong) for library preparation and sequencing. 100 bp paired end RNA-seq libraries were prepared according to the BGISEQ-500 RNA-Seq Library Preparation Protocol and libraries were sequenced on a BGISEQ500 sequencer (Beijing Genomics Institute).

For linkage mapping, 2 plants were grown from seed collected from populations (i) WWA-C and (ii) WWA-M (Papadopulos et al. 2021) and were subject to a controlled cross. One of the offspring of this cross was selfed and the resulting seeds (F2s) collected (Supplementary note 2). When the plants were 8 mo old, leaf punches were taken for DNA extraction and sequencing using LGC Genomics GmbH SeqSNP service. LGC Genomics constructed SeqSNP sequencing libraries for F2 progeny using 25,000 custom probes (Supplementary Table 1). These libraries were sequenced on an Illumina NextSeq 500.

### Genome size estimation

The nuclear DNA content of *Silene uniflora* was determined by flow cytometry following the 1-step procedure described in Doležel et al. (2007); see Supplementary note 3.

### Genome assembly

An initial draft hybrid assembly was constructed from raw Illumina short read data and PacBio longread data using the MaSuRCA pipeline (kmer size = 99; PacBio N50 = 6,149 bp; PacBio mean sequencing depth = 29.21x; Supplementary Table 2). Contigs from this draft assembly were then arranged into scaffolds using the Hi-C and Chicago data using the Dovetail HiRise pipeline. To further improve the assembly, we utilized *Oxford Nanopore MinION* (ONT) long reads. ONT data (N50 = 11,254 bp; mean sequencing depth = 12.55x; Supplementary Table 2) were first corrected using the short reads produced from the Hi-C scaffolding (Supplementary note 4). Corrected long reads were first used to perform additional scaffolding of the assembly before being used to close assembly gaps. Scaffolding was conducted using SLR v1.0.0 (Luo et al. 2019) with a minimum alignment length of 300 nucleotides. Gap closing was conducted using *TGS-GapCloser* v0.56 (Xu et al. 2020). Contamination was assessed using the NCBI Foreign Contamination Screen pipeline (Astashyn et al. 2024) and any adaptor and non-target organism sequences were masked from the final assembly. For each step in assembly improvement, QUAST (Gurevich et al. 2013) was used to assess assembly size and contiguity. To further assess assembly contiguity, we calculated the LTR Assembly Index (LAI) using LTR retriever v2.9.0 (Ou et al. 2018; Ou and Jiang 2018).

### Genome annotation

Prior to gene prediction, we masked repeat regions using *RepeatMasker* (v4.1.0; http://www.repeatmasker.org/) against the MIPS plant repeat database (v.9.3; (Nussbaumer et al. 2013). For gene annotation, we employed RNA-seq mapping, assembled transcript mapping, protein mapping and *ab initio* approaches, which were then combined into a single non-redundant gene model (Supplementary note 5). We used eggnog-mapper v2.0.1 with database version 2.0 (Cantalapiedra et al. 2021) to functionally annotate predicted proteins. We used diamond as a mapper, a minimum query and subject coverage of 70%, an e-value cutoff of 0.0001, a taxonomic scope of Viridiplantae and defaults for all other options. To produce a final set of high-quality gene annotations, we filtered out annotations with non-canonical splice sites or in-frame stop codons, those annotated as transposable elements in eggnog-mapper, those without start or terminal stop codons, and those without either RNA evidence or matches to know ortholog groups in eggnog-mapper. Annotation completeness was assessed using BUSCO v4 (Simão et al. 2015) in protein mode with the Viridiplantae, Embryophyta, and Eudicot gene sets. To estimate the position of centromeres in the 12 largest scaffolds, we used CentIER v2.0 (Xu et al. 2024) with our final gene annotation and default settings. To identify telomeres within the 12 largest scaffolds, we used quartet v1.2.5 (Lin et al. 2023).

### Linkage map construction and analysis

Raw reads from the SeqSNP data were trimmed and mapped to the genome (Supplementary note 6). Linkage maps were constructed using LepMap3 v0.4 (Rastas 2017); see Supplementary note 7. The linkage map was used to provide validation of the genome assembly, indicate the position of unassigned scaffolds, and estimate local recombination rate. The mapping parents originated from different populations to the reference individual, so structural variation between populations could introduce misassemblies. Therefore, the linkage map was not used for scaffolding. Recombination rate was estimated using a LOESS local regression (span 0.1 Mb), with recombination rate estimated as the slope in non-overlapping 1 Mb windows. To avoid inaccurate recombination rate estimates caused by error or rearrangements between the plants used for genome assembly and linkage mapping, we did not calculate recombination rate for windows containing no markers or where the map window contained markers from other scaffolds.

### Comparative genomics

Synteny between the *S. uniflora* genome and the previously published assemblies of *S. conica* and *S. latifolia* was examined using the MCscan algorithm implemented in the JCVI toolkit v.1.3.8 (Wang et al. 2012; Tang et al. 2024). Previously identified sex-linked transcripts from *S. otites*, *S. pseudotites,* and *S. latifolia* (Chibalina and Filatov 2011; Zemp et al. 2016; Martin et al. 2019) were mapped to the genome using minimap2 v.2.24-r1122 (Supplementary note 8).

## Results and discussion

The mean estimated genome size from our flow-cytometry analysis was 1C = 1.28 pg, corresponding to a haploid genome size of 1251.48 Mb (Supplementary Table 3). The final genome assembly was 1269.36 Mb long, closely matching the estimated genome size. The assembly contained 12,573 scaffolds, had a scaffold N50 of 40.72 Mb and a scaffold L50 of 11. Approximately 53.73% of the assembly was contained within the 12 largest scaffolds which were between 32.17 and 76.86 Mb in size (henceforth chromosomes Chr01–Chr12), corresponding to the haploid chromosome number of *S. uniflora*. Additionally, 5 further scaffolds were over 1 Mb in length. Contamination screening identified no foreign organism sequences and 5 putative adaptor sequences (totaling 134 bp; Supplementary Table 4) which were masked from subsequent analysis. Starting from an initial assembly with an N50 of 34 Kb, each step in the genome assembly significantly improved genome contiguity (N50 values: Chicago: 169 Kb; Dovetail Hi-C: 27.01 Mb; ONT scaffolding: 34.97 Mb, ONT gap-filling: 40.72 Mb; Supplementary Table 5). The genome contains ∼29% repetitive sequences, with LTR retrotransposons contributing the largest fraction (∼25%; Supplementary Table 6). The assembly had a raw LAI score of 10.29 and a corrected LAI score of 4.66. The final gene annotation contained 41,603 protein coding genes encoding 48,596 putative transcripts. Of these, 98.89% were complete, including both start and stop codons and 32.26% also contained annotations for 5′ and 3′ untranslated regions (Supplementary Fig. 1). BUSCO analysis suggested that the annotation was largely complete (86% to 91% BUSCO completeness depending on the gene set used; Supplementary Fig. 2).

The linkage map contained 8,372 markers on 1,357 scaffolds which were assigned to 12 linkage groups (LGs; Supplementary Table 7). The linkage map was used to validate the assembly of the chromosomes and estimate the correct placement of other large scaffolds.

**Fig. 1.**
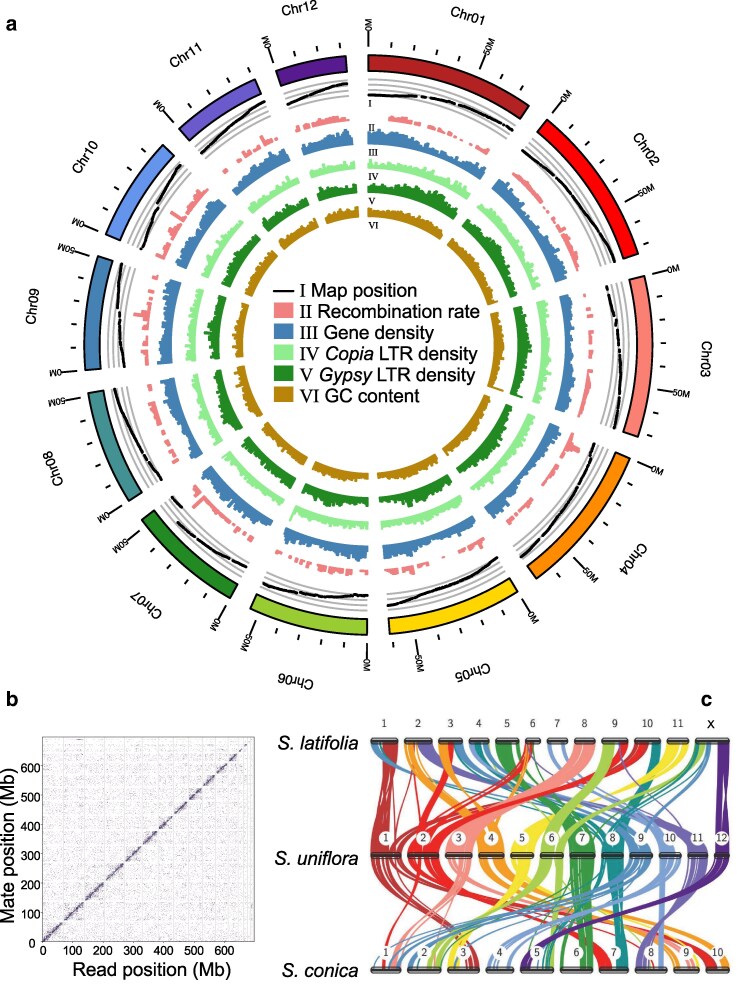
Genome assembly of *Silene uniflora* and comparison to other *Silene* genomes. The *S. uniflora* chromosomes (ie the twelve largest scaffolds) are shown as a circular plot (a) with GC content (%), *gypsy* and *copia* LTR retrotransposon density, gene density (genes per 1 Mb), and estimated recombination rate (cM/Mb) shown in non-overlapping 1 Mb windows. The relationship between physical and genetic distance is shown as a “Marey map” (Chakravarti 1991), with each point representing one genome-anchored marker and the *y*-axis representing its position in our linkage map. The Hi-C scaffolding is shown as a link density histogram (b) with the genomic position of each read and its mate shown on the *x* and *y* axes, respectively and color intensity representing number of read-pairs per bin. Comparison of the *S. uniflora* genome with previously published *Silene* genomes shows widespread rearrangements between the species (c). Links between the chromosomes of each species represent syntenic blocks shared between the species, colored by *S. uniflora* chromosome as in panel A.

LGs were broadly concordant with assembled chromosomes. Between 87.2 and 97.5% (mean = 95.6) of markers from each chromosome were assigned to a single LG, and the LG differed for each chromosome (Supplementary Table 8). The assembled sequence for ten of the chromosomes covered over 90% of the map length of its corresponding LG (Supplementary Table 8). This value was lower for Chr11 and Chr12 (58 and 79% respectively; Supplementary Table 8), and the map suggested that 3 of the 5 unplaced scaffolds >1 Mb were linked to Chr12 in LG.4 (Supplementary Fig. 3) and one to Chr11 in LG.1 (Supplementary Fig. 4). The final unplaced scaffold >1 Mb was linked to Chr02 in LG.3 and embedded within Chr02 in the map (Supplementary Fig. 5). One putative centromere location was identified for all chromosomes except Chr01, for which 2 were identified. We did not identify telomeres in any chromosomes. Regions of high repeat density and low gene density were found toward the center of several chromosomes (Fig. 1; Supplementary Fig. 6), and these corresponded to centromere locations estimated by CentIER for these chromosomes (Supplementary Fig. 7). Therefore, evidence from linkage map-genome concordance and gene and repeat distribution indicate that, while this is not a telomere-to-telomere assembly, the majority of the assembled chromosomes are largely complete, with the exceptions of Chr11 and Chr12.

Some inconsistencies between the map and assembly may represent rearrangements between the populations that the genome and mapping plants originated from. For example, plots of physical vs map position highlight a putative intrachromosomal translocation on chromosome Chr07, and putative inversions on Chr01, Chr03, Chr06, Chr09, and Chr10 (Supplementary Fig. 8). Frequent chromosomal rearrangements may have important implications in *S. uniflora*, since these have the potential to contribute to reproductive isolation between locally adapted populations (Kirkpatrick and Barton 2006).

Mean local recombination rate varied between chromosomes (ranging from 1.35–2.12 cM/Mb; Supplementary Fig. 9) and was negatively correlated with chromosome length (Spearman's rank correlation: *ρ* = −0.87; *P* = 0.0004; Supplementary Fig. 10), a common finding across eukaryote species (Haenel et al. 2018). Within chromosomes, and on a genome-wide scale, local recombination rate was significantly positively associated with gene density, but not GC content, despite gene density and GC content being correlated with each other (Supplementary Fig. 11). *Gypsy* and *Copia* LTR retrotransposon density were both significantly negatively associated with gene density (Supplementary Fig. 10).

Comparison to the *S. latifolia* and *S. conica* genomes revealed widespread rearrangements between the species. Notably, the X chromosome of *S. latifolia* was linked to 4 *S. uniflora* chromosomes. It has previously been suggested that chromosomal translocations have played a causative role in sex chromosome formation in *S. latifolia* and its sister species *S. dioica* (Bačovský et al. 2020). However, our synteny analysis suggests that most large-scale rearrangements in the *S. latifolia* X chromosome relative to *S. uniflora* are shared by *S. conica*—a hermaphrodite lacking sex chromosomes—suggesting these rearrangements occurred prior to the evolution of dioecy (Fig. 1c). Sex chromosomes have evolved independently in multiple *Silene* lineages. We found that previously identified sex-linked transcripts from *S. latifolia*, *S. otites* and *S. pseudotites* all mapped to different chromosomes in the *S. uniflora* genome (Supplementary Fig. 12), confirming previous evidence that sex chromosomes have evolved from different autosomes in each of these 3 species (Martin et al. 2019).

Here, we present a high-quality genome assembly and linkage map for *S. uniflora*, which we use to infer the recombination landscape of *S. uniflora* and investigate genome rearrangements between *Silene* species. This will be an important resource for studies using *Silene* as a model organism. *S. uniflora* populations have repeatedly and rapidly evolved tolerance to heavy metal contamination. A high-quality genome assembly will allow the role of genomic rearrangements in this process to be examined. Furthermore, as one of only 3 *Silene* species with a high-quality genome assembly, this resource is likely to be important for studies of other species and Caryophyllaceae more broadly.

## Supplementary Material

jkag002_Supplementary_Data

## Data Availability

Assembly, annotation and the raw data used is accessible under the NCBI project PRJNA800752. Raw data used for the linkage map is accessible under the NCBI project PRJNA764594. All code used in the analysis is available at https://github.com/ogosborne/Silene.uniflora.genome.project. Supplemental material available at G3 online.
